# Dignity in mental health care: the conceptual connections between sense of dignity, stigma and self-stigma among people with mental disorder diagnoses

**DOI:** 10.3389/fpsyt.2026.1600777

**Published:** 2026-02-02

**Authors:** Kamila Sap, Radosław Stupak

**Affiliations:** 1 Institute of Psychology, University of the National Education Commission, Krakow, Poland; 2 Institute of Psychology, Cardinal Stefan Wyszyński University in Warsaw, Warsaw, Poland

**Keywords:** dignity, mental disorder, mental health, self-stigma, sense of dignity, stigma

## 1 Introduction

Exactly what is dignity? The concept is contemplated in sociology, theology, law, health care, pedagogy, philosophy, psychology and psychiatry (1–6), with different disciplines contributing their specific valuable perspectives and understandings, making the phenomenon multi-faceted and not easy to pin-down. Dignity, understood as an attribute of every human being, is described as an unquestionable and inviolable value deriving from being human, forming the basis of equality for all people (7). Dignity, considered ontologically, describes a person regardless of the situation the person finds herself in and it cannot be shattered. From this perspective, dignity is inherent to every human being, emphasizing that no person should be treated as a means to an end or as a commodity (8, 9). Dignity is also an important part of human rights (10). Here, we would like to focus on the concept of dignity from the perspective of mental health, psychiatry and psychology. Within these fields dignity is described as a practical and crucial element of effective and ethical mental health care, which is essential for well-being, while threats to dignity can be linked to poorer mental health (11). For instance, among psychiatric inpatients, a diminished sense of dignity was associated with increased clinical severity, reflected by both past suicide attempts and present suicidal thoughts (12).

The theory of Public Health assumes that lack of dignity is associated with an increased risk of disease (13). Public health should recognize dignity (and thus a sense of dignity) as one of the core values and an element of human well-being. Respect for dignity should be an important principle guiding health interventions (14–16). Proposing an ethics framework for public health, Kass (17) emphasized that ethical guidelines for clinical practice, while maintaining the imperative to avoid harm, began explicitly requiring the preservation of patient and research participant dignity. Insufficient recognition of human dignity in healthcare can provoke adverse emotional responses – including fear, distrust, denial, anger, hostility, apathy, sadness, and frustration – which, in turn, have an unquestionable impact on patients’ overall health (18). The World Health Organization (19) emphasizes its commitment to ensuring that individuals with mental health conditions are able to lead lives characterized by dignity. In this regard, WHO (20) launched various initiatives, which aimed to support countries in developing high-quality mental health services that respected the dignity and autonomy of patients and supported recovery. Therefore, dignity should be considered a fundamental value in psychiatry.

If dignity is considered the foundation of humanity, social relationships and well-being (21), it is all the more appropriate to raise this issue among people with mental disorder diagnoses, as they have been and/or may be at risk of discrimination (22), face difficulty getting a job (23), poverty (24), domestic violence (25), sexual abuse (26), involuntary hospitalization (27), stigmatizing attitudes among psychiatrists (28) and clinical psychologists (29). Additionally, the mental health sector can also reinforce stigmatizing views and attitudes toward people with lived experiences of mental disorder diagnoses (30). One such form of discrimination can be diagnostic overshadowing, which occurs when physical symptoms are incorrectly attributed to mental health conditions, leading to missed or inaccurate diagnoses (31). Patients reports suggest that the physical health needs of individuals with mental disorder diagnosis are often not given adequate attention by healthcare providers (32). This is particularly evident in patients with complex presentations, where the coexistence of psychiatric and somatic symptoms frequently leads clinicians to prioritize psychiatric explanations within the differential diagnosis (33). The consequences are increased distress, limited medical treatment, and even death (34, 35). Thus, the institutional reality of mental health care may be particularly harmful to the sense of autonomy, respect, and dignity. Some authors emphasize that the mere experience of psychiatric treatment – including contact with a psychiatrist, the use of medications that may cause side effects, and the often inadequate standards of psychiatric hospitals – can be stigmatizing for patients (1). On this account, these experiences are discussed within the context of dignity in psychiatric care (1).

In this article, we focus on individuals with mental disorder diagnoses, however, it is important to note that stigmatization or threats to dignity affect other groups as well, including sexual minorities (36), racial minorities (37), individuals living with AIDS/HIV (38), children in foster carer (39) and others.

Although dignity is an attribute of every human being, a sense of dignity can be gained or lost in interaction with other people (40). The studies that describe experiences of dignity of individuals confirm that a person’s sense of self-dignity can be undermined, particularly when individuals feel their ability to decide for themselves is limited and autonomy is restricted, or when they lose the ability to function independently (41–43).

One of the few studies that examined the sense of dignity in people with a diagnosis of a mental disorder, specifically people with a diagnosis of schizophrenia, highlighted connections between the issues of stigma and self-dignity. The results suggested that stigma and discrimination can erode the sense of dignity, particularly among older people (44, 45). People taking part in the survey said that a diagnosis of schizophrenia and the stereotypes associated with it make it difficult to experience dignity. Additionally, statements from some people indicated that they believed they did not deserve to experience dignity (44). This may indicate an internalization of negative beliefs and attitudes (46). The labeling of a person as a “person with schizophrenia” may be destructive in itself (47). Sometimes this kind of labeling can cause more problems than the issues called symptoms of schizophrenia (48) or depression (49). Seikkula (50) (p. 5), one of the authors of Open Dialogue wrote: “I receive emails every day from patients and from family members all over the world and they repeat the message: ‘No one listens to me’. No one takes it seriously.”

People with diagnoses might feel misunderstood, isolated, ignored. Over the years, the lack of respect for human dignity in psychiatric hospitals may have contributed to the stigmatization of people with mental disorder diagnoses (51).

Admittedly, the issue of stigma has been widely discussed (52–54), which is reflected in declarations such as: “Ending mental-health-related stigma is a goal that must be pursued” [ (55), p. 1381] and stigma is recognized as a public health problem (56). On the other hand, the issue of dignity, and especially the sense of dignity of people with mental disorder diagnoses is often neglected, even though psychiatric patients may face particularly strong threats to dignity (57). This may be related to the persistence of stigmatization and, consequently, self-stigmatization.

The dignity of people with a diagnosis is sometimes recognized and discussed, but research on this topic is scarce. It has been reported, though, that a lack of sense of dignity among patients in psychiatric wards may be related to higher perceived coercion, better insight, and more negative symptoms (58, 59). This contrasts with the treatment of individuals with somatic illnesses, terminal illnesses, or the elderly, as the topic and importance of a sense of dignity is addressed in many studies (41, 42, 60–66).

We believe that reflection on the concept of dignity, followed by the development of appropriate tools to measure dignity in the context of mental health and psychiatry is essential. This is particularly important given that, despite many mental health awareness campaigns and the growing public engagement with mental health topics (67, 68), as well as the popularity of social media accounts educating about mental disorders (69, 70), stigmatization – especially of the so-called serious mental disorders – persists, fueling self-stigmatization (53, 54, 71) and potentially undermining individuals’ sense of self-dignity.

If sense of dignity can be conceptualized as a resource that protects against harm (72) then, can it protect against internalizing stigma? A related question concerns understanding the sense of dignity. How is the sense of dignity understood, how can it be operationalized, what should constitute this construct, and will it not be understood as simply the opposite of self-stigmatization?

## 2 Methodology and purpose

The aim of our article is to provide a reflection on the concepts of stigma, self-stigma, dignity and sense of dignity, and to explore their interrelations, particularly the relationship between self-stigma and the sense of dignity. Since these problems seem to be understudied, both conceptually and empirically, we chose a methodology that allows for flexibility, and the integration of insights from diverse sources. We adopted a critical review approach, which combines elements of a narrative review and conceptual analysis. This is particularly suited to our goal, because it enables us to examine concepts in a narrative way, rather than to synthesize existing empirical findings in a strictly systematic manner.

According to the typology of Grant and Booth (73), narrative reviews are typically based on existing publications and do not always aim to exhaustively search all possible sources or rigorously evaluate their methodological quality. They are more flexible than systematic reviews (74), and do not require fixed inclusion or exclusion criteria (75). This flexibility is essential for our conceptual paper and aligns with the configurative orientation adopted here, which prioritizes identifying a sufficient range of relevant sources to examine patterns and concepts rather than striving for comprehensive coverage (76).

We recognize that this approach has several limitations: it does not aim for exhaustive coverage of the literature, and the purposive nature of our search, without rigid inclusion or exclusion criteria, can introduce selection bias (73). However, we have strived to minimize these risks by using diverse sources, drawing upon theoretical papers as well as both quantitative and qualitative studies, and theoretical papers in order to include the most relevant and significant research to the topic. Most importantly, despite these limitations, this method is appropriate for our objective – developing a theoretical account of how the concepts of dignity and self-stigma intersect and/or differ in the context of mental health care.

## 3 Stigma

Stigmatization as a concept was introduced to the social sciences by Goffman (77). He defined stigma as a deeply discrediting trait, although he emphasized that the language of relationships rather than attributes was more suitable for describing stigmatization (77). According to Corrigan and Watson (78) public stigma has three core components: stereotypes, prejudice, and discrimination. In the case of public stigma, stereotypes refer to negative beliefs about a particular group – for example, perceptions of danger, weakness, or incompetence. Prejudice occurs when individuals accept these stereotypes as accurate and/or believe it is acceptable to feel certain emotions toward the group, such as fear or anger (78). Discrimination, the third element, involves behavioral responses such as avoiding members of the stigmatized group. However, stigmatization can be also structural (79), meaning “societal-level conditions, cultural norms, and institutional policies that constrain the opportunities, resources, and wellbeing of the stigmatized” [ (80), p. 2]. The concept of structural stigma is rooted in conceptualizations of institutional racism (81). Early discussions of structural stigma often referenced examples of structural racism and racial discrimination, such as Jim Crow laws (82). Structural stigma is linked to the discrimination of social groups at the institutional, legal, and societal levels, contributing to inequalities, including disparities in health (83). This embodiment of public stigma in official institutional regulations and practices may be one of the conceptual differences between public stigma, defined mostly at the level of beliefs and individual actions, and structural stigma, referring more to the policies, organization and institutions of the society.

One of the most well-known theories of stigma was proposed by Link and Phelan (84). In their theory, the stigmatization process consists of labeling, stereotyping, categorization, loss of status, and discrimination. Furthermore, emotional reactions play an important role and are crucial to understanding the behavior of both those who stigmatize and those who are stigmatized (85). A mental disorder diagnosis can function as a label which, like other labels, is not self-evident but is socially constructed (86). Moreover, the presence of psychopathological symptoms is not always necessary for an individual to be assigned such a label (86). Diagnosis is closely linked to stereotyping, as the clinician – by assigning a person to a particular diagnostic category – tends to overlook individual characteristics and instead seeks similarities between the person and the symptom profile of the given disorder (86, 87). Diagnostic practices also exemplify categorization, whereby individuals with psychiatric diagnoses may be perceived as a group distinct from others, while simultaneously being regarded as homogeneous and possessing fixed traits (86, 87). In this case, individuals diagnosed with a mental disorder are exposed to status loss and discrimination. This may be reflected in some of psychiatrists’ attitudes, for example, in a Polish survey, 76% of surveyed psychiatrists stated they would not accept a “mentally ill” person to provide childcare for their offspring, and 51% that they would not want such a person to become their daughter-in-law or son-in-law (28).

Psychiatric diagnostic labels share the features described by Link and Phelan: they are socially constructed, relative, simplified, sometimes arbitrary, and potentially inaccurate (86). However, stigmatization can arise not only from an official diagnosis but also from self-labeling, that is identifying oneself as a person with a mental disorder label (49). Modified labeling theory proposes that (self-)labeling of mental disorders can harm individuals through two routes: directly, by internalizing labels and lowering self-concept, and indirectly, through maladaptive coping strategies in response to the stigma (88). In spite of the fact that self-labeling may have positive outcomes (e.g. facilitate help-seeking), it can be associated with perceived lower control over depression (49). Therefore, not only official diagnoses but also self-labeling may play a role in the context of stigma, self-stigma and dignity, as diagnostic labels can become salient and persist, influencing how behavior is interpreted or, in the case of self-labeling, self-concept and how people perceive themselves, potentially leading to, for example, maladaptive coping strategies (49).

The level of stigma is still high, especially for the so-called serious mental disorders, which include schizophrenia (53, 54, 89). In Germany, stigma associated with a schizophrenia diagnosis increased over the last 30 years (1990–2020), while it decreased marginally for a diagnosis of depression (54). The study employed a repeated cross-sectional design using representative population samples from the western federal states of Germany. Face-to-face surveys were conducted in 1990 (n = 2044), 2001 (n = 4005), 2011 (n = 1984), and 2020 (n = 2449). Participants were provided with a diagnostically unlabeled vignette matching DSM-III-R criteria for schizophrenia or depression. Stigma was measured using the Social Distance Scale and the Emotional Reactions toward Mental Illness Scale. For schizophrenia, social distance increased in six of the seven assessed situations (e.g., renting 203 a room, work recommendation), and did not change in one (marriage into the family). Negative emotional reactions, such as fear and feelings of anxiety, also increased, while the willingness to offer help declined. For depression, social distance decreased in two situations (marriage into the family, renting a room) and increased in one (childcare), while remaining largely unchanged in the remaining situations. The authors acknowledged several limitations of their study, including the focus on major depression and schizophrenia, the restriction to the population of West Germany, and the declining response rates over time – from 70% in 1990 to 57% in 2020 – though such a decline is typical in longitudinal survey research. Pandemic restrictions may have also contributed to that decline and the fact that respondents could fill the survey by themselves in 2020, while the interviewer waited outside, could have influenced the results, for example by lowering the social expectations effect. Despite these limitations, the study remains an important contribution to stigma research indicating that, in spite of efforts, stigmatization persists or even increases.

At the same time, due to public education efforts in Germany, the number of people using medical language to describe individuals and correctly applying diagnostic criteria for schizophrenia and depression increased; however, the use of derogatory terms, such as “crazy” or “insane”, remained at fairly stable levels over the years (90), suggesting that medical labeling and derogatory labeling are not mutually exclusive. Notably, these temporary increases in derogatory labeling – such as in 2001 – may reflect the influence of the 1990s “Decade of the Brain”, a period characterized by heightened emphasis on biomedical explanations and the dominance of biogenetic models of mental disorders (90, 91). On the other hand, there was a slight decrease in clearly derogatory labels and an increase in the use of “trivializing” terms (such as “should have a holiday”, “has no responsibilities”, or “should sleep in”) for symptoms of depression (90). Hazell et al. (92) proposed a kind of “stigma hierarchy” and confirmed that the diagnoses associated with the greatest stigma were schizophrenia and antisocial personality disorder, while the less stigmatizing diagnoses were depression, generalized anxiety disorder, and obsessive-compulsive disorder. Some studies also indicate that stigmatization of people with a bipolar disorder diagnosis remains at a similar level to people with a schizophrenia diagnosis, but other studies contradict this (93). The discrepancies in the results may have different reasons, while the context in which the research is conducted may play a crucial role (93).

Stigma may be most severe for people with schizophrenia diagnosis. In the United States, people with a diagnosis of schizophrenia were more likely to be perceived as potentially dangerous or violent in 2018 than in 1996 and there was a higher public support for involuntary hospitalization (94). According to some patients, the schizophrenia label resulted in “feeling utterly worthless and defective” [ (95), Theme 2: Stigma of diagnosis and selective disclosure] so any other label would have been better: “bipolar diagnosis is better than getting anything with the word schizo in it” [ (95), Theme 2: Stigma of diagnosis and selective disclosure]. A qualitative study on the stigma of mental disorders in the context of motherhood found that mothers want to reject the label of a “crazy woman” and distinguish between diagnoses that are less and more “harmful” (96). Indeed, one woman stressed that her diagnosis (post-traumatic stress syndrome) “was not as bad as something worse would have been in the end, like schizophrenia or borderline [personality disorder], or … deep manic-depressive phases or something like that” [ (96), p. 9]. Distancing oneself from schizophrenia diagnosis can be related to beliefs about incurability, biological causes, dangerousness, unpredictability (97). Professionals can also be more stigmatizing and less positive, for instance, about people with a schizophrenia diagnosis than those with a depression or anxiety disorder diagnosis (98). Social stigma may not only be correlated with self-stigmatization, but may prospectively predict the occurrence of self-stigmatization which may suggest the casual nature of social stigma (99).

## 4 Self-stigma

Internalization of stigma is a subjective process embedded in the socio-cultural context. It can be characterized by negative feelings about oneself, maladaptive behavior, an attempt to change one’s identity or the endorsement of a stereotype (100). Self-stigmatization could be described as a process consisting of several stages. According to Corrigan and Rao (46), the model of self-stigmatization that applies to people with diagnoses of mental disorders has the following stages: first, a person is aware that society may judge people with diagnoses negatively, next they begin to accept these negative beliefs as a general truth, and finally accept it as a truth about oneself and internalize a belief about, for example, being worthless. In summary, self-stigmatization involves agreeing with stereotypes about oneself (e.g., “I’m dangerous”), prejudices (e.g., “I’m afraid of myself”), and the resulting discrimination (e.g., isolating oneself) [ (46), p. 465]. This, in turn, causes a decrease in self-esteem and self-efficacy (101).

Several models of internalized stigma can be distinguished (102). The socio-cognitive model assumes that awareness, acceptance and use of stereotypes about oneself cause devaluation, inability to set life goals, depression, and negative attitudes toward recovery, in addition, stigma damages individuals’ self-esteem (22, 102, 103). The Model of Personal Discrimination, Internalized Stigma, and Intention to Participate in Collective Actions indicates that personal discrimination (discrimination directed against oneself for belonging to a stigmatized group) predicts a higher internalization of stigma in people with mental disorder diagnoses, while group discrimination (discrimination perpetrated by others against a stigmatized group) predicts higher willingness to participate in collective actions, such as protests or social movements (102, 104). The socio-cognitive-behavioral model, which has been partially confirmed by research, assumes that the way people experience mental disorders depends on their attitude toward the disorder and previous experiences of discrimination (102, 105).

Recently, studies have shown that internalized stigma does not need to be moderate or severe to have negative consequences; even mild levels of self-stigmatization can lead to impaired functioning for people with a diagnosis of mental disorder (106). Self-stigma is related to lower self-esteem among individuals with schizophrenia diagnosis (r = -0.758, p < 0,01) (107, 108), and among people with several other mental disorder diagnoses (r = -0.56, p < 0,001), lower self-efficacy (r = -0.54, p < 0,001), worse well-being and satisfaction with life (85), lower hope (r = -0.58, p < 0,001) and less empowerment (r = -0.52, p < 0,001) (100), higher suicidal ideation (r = -0.41, p < 0,001) (109), reluctance to seek help from psychiatrists and general practitioners (but not from psychologists or informal help) (110), abandonment of treatment (111), depressive symptoms (r = 0.41, p = 0,001) (112), social self-worth contingencies (r = 0.29, p < 0,01) (113), reduced self-respect and stronger “why try” effect (114), negative past and future time perspective (115), negative association with subjective social status (r = -0.21, p = 0,002) (116), worse quality of life (r = -0.57, p < 0,01) (117), and help-seeking (118). Overall, the reported effect sizes of the associations between self-stigma and negative outcomes range from moderate to large (r = 0.29 – 0.76). However, most studies are cross-sectional and questionnaire-based, using a variety of measures and samples, which limits the possibility of causal inferences comparability across studies and generalization of the results. Still, the reported associations cannot be ignored and point to possible serious consequences in social functioning, for example in one study self-stigma was negatively correlated with academic and social functioning in late adolescence (119). Additionally, people who experienced stigma said they were blamed for having a diagnosis of a mental disorder as the disorder may be perceived by others as a failure that can be avoided or overcome at any time (113).

Although self-stigma can significantly undermine well-being, functioning, and coping, these negative consequences highlight the importance of strategies aimed at reducing self-stigma and promoting sense of dignity.

## 5 Sense of dignity

Dignity, even though sometimes invoked in the mental health care discourse, is rarely addressed in empirical research as a sense of self-dignity, which could be explained by the perceived ambiguity of the concept. Therefore, we decided to focus on selected concepts of dignity. Staats et al. (43) (pp. 5-6) pointed out that, contrary to dignity understood ontologically:

“Relative dignity is (…) a modifiable form of dignity that is influenced by sociocultural factors of everyday life. Relative dignity concerns feelings of self-worth as well as worthiness in relation to other people. It can be strengthened through the support and confirmation of others, but can also be torn down and violated.”

A sense of dignity is a way of experiencing being a worthy and complete human being. Rumiński (120) wrote about the sense of dignity as a kind of positive opinion about oneself. Loss of this sense could result in self-condemnation. However, in contrast to self-esteem, it does not foster an instrumental approach to life (121). The sense of dignity can be socially determined – people’s self-respect is based on whether they receive this respect from other people (122). Treating someone with dignity can include supporting their autonomy (42) or acting honestly (123).

Jacobson (124) proposes a taxonomy of social dignity consisting of dignity-of-self and dignity-in-relation. Dignity-of-self refers to self-respect and self-worth, characterized by confidence, integrity, and a dignified demeanor. Dignity-in-relation involves showing respect and worth through personal and collective behavior and includes the historical connection of dignity to social status or rank (124). The expectations and perceptions of dignity are shaped by the customs and traditions of a society. Since dignity is socially constructed, it can be assessed, violated, or promoted. Jacobson (124) identifies three core aspects of dignity:

1. Dignity Encounters – Each and every interaction with another individual represents an occasion to experience dignity. An encounter may be either public or private, yet it is invariably situated within a social framework. In the context of social interaction, the dignity of an individual or group can be either upheld or undermined. The violation of dignity is more likely to occur when one of the individuals is in a state of illness, financial deprivation, helplessness, or shame, and the other displays a lack of acceptance or prejudice. Furthermore, violations are more likely to occur in asymmetrical relations, wherein one side possesses greater knowledge, power, material resources, or strength. Conversely, interactions grounded in openness, empathy, respect, and trust are more conducive to the promotion of dignity. It is noteworthy that the experience of dignity is instrumental to the advancement of social equity (124).

“Finally, dignity promotion is more likely to occur under an order of justice, a social order that sees the provision of adequate income and housing, access to education and healthcare, and other societal investment in public goods” [ (124), Forms of dignity].

2. Dignity Violation – These are attitudes that may be perceived as being discourteous, condescending, indifferent, contemptuous, objectifying, categorizing, labeling, mistrustful, exploitative, or coercive (124).

3. Dignity Promotion – The characteristics in this category include: being independent, fulfilling everyday duties, supporting others, appreciating others, politeness, symmetrical relationships, creativity (124).

Kozielecki (125), a Polish psychologist, believed that a sense of dignity is consolidated through activity. In this approach, dignity is seen as three-dimensional and depends on actions such as defending one’s own identity and belief system, activity directed toward other people (solidarity with others, emotional ties), and creativity. Faithfulness to oneself, taking up non-personal goals, and creative activity are the three main sources that develop an individual’s belief in their worth as a human being (125). According to Kozielecki, non-personal goals, include, for instance, commitment, altruism, amity (125). In addition, Kozielecki (126) claimed that as in any self-assessment, which is a judgment about oneself and one’s behavior, the sense of dignity plays a role in an individual’s life: it affects the individual’s well-being and actions.

Among other characteristics or kinds of dignity, Nordenfelt identified the ‘Dignity of Identity’, describing it as, “quite difficult to define but is probably the most important kind in the context of illness and ageing. It is significant of this kind of dignity that it can be taken from us by external events, by the acts of other people as well as by injury, illness and old age” [ (127), p. 26].

While individuals may have basic respect for each other, this dignity can be eroded by “cruel acts of other people” [ (127), p. 26]. In addition, the important factors underlying the dignity of identity are the integrity and autonomy of the subject, including social relations of the person (127).

It seems that the common thread of these theories is the social component of the sense of dignity, meaning that it is in interactions with others that we can affirm and strengthen it, but also experience loss of dignity. Having a sense of dignity means self-acceptance, self-respect, and self-confidence, as well as showing respect for others and receiving it from them (128). Theories of dignity stress the importance of personal autonomy, which seems to be one of the elements of having a sense of dignity (129, 130). Jacobson and Silva (131) point out, however, that neither autonomy nor self-esteem is merely a straightforward sum of dignity, nor can dignity be reduced solely to these components. In addition, a sense of dignity is created in action, including activity directed toward other people (125), so it can preclude social isolation and feelings of alienation (44).

## 6 Self-stigma and a sense of dignity

Our research focuses on the issue of self-stigma and sense of dignity, particularly in the context of people with mental disorder diagnoses. We have not found studies that have thoroughly examined internalized stigma and sense of dignity among groups labeled as mentally disordered and especially vulnerable to stigma. It is interesting to consider how the concepts may intersect and to what extent they may be construed as opposite or distinct (**Table 1**).

**Table 1:** Sense of dignity, self stigma, public stigma and structural stigma.

Sense of dignity			Self-stigma	Public stigma	Structural stigma
Sense of dignity involves experiencing oneself as a worthy and complete human being, being acknowledged as such by others, and being able to act autonomously in line with one’s own values.			Self-stigma can be characterized by negative feelings about oneself, maladaptive behavior, or the endorsement of stereotypes.	Public stigma refers to negative attitudes and beliefs held by the general public that lead to fear, rejection, avoidance, and discrimination toward a stigmatized group. Public stigma has three core components: stereotypes, prejudice, and discrimination.	Structural stigma is linked to the discrimination of social groups at the institutional, legal, and societal levels, contributing to inequality.
Theme and Subtheme	Promotion	Violation			
Autonomy 1. Privacy. 2. Freedom of choice or movement.	Involvement in care decisions through shared decision-making. Beneficent action by healthcare professionals through supporting a patient’s sense of self and moral agency, including avoidance of pressure to initiate or continue treatment (131).	Coercion and involuntary psychiatric care (59). Informal/soft coercion (pressure/manipulation, threats, deception). It may be applied by mental health professionals, but also by the person’s family (132).	Among individuals with a history of involuntary hospitalization: higher baseline shame, self-contempt, and stigma-related stress – associated with greater self-stigma and lower empowerment at 1-year follow-up (133).	Public stigma as a significant predictor of approval of coercion, particularly perceived dangerousness – the belief that individuals with mental disorders are unreliable, unpredictable, and potentially harmful – associated with greater overall endorsement of coercive measures (134). Support, especially for involuntary hospitalization, was higher when individuals were perceived as less capable of making treatment decisions and as posing a risk to themselves or others (135).	Reported experiences among individuals with mental health-related diagnoses of punitive or coercive care and non-person-centered care, both linked to structural stigma (136). Potential lasting effects of involuntary treatment, including reduced trust in mental health services. Asymmetrical power dynamics, physician dominance, patronizing clinician attitudes, limited willingness to include patients in decision-making, limited awareness of available choices, and failure to recognize the option to refuse treatment (137).
Acceptance and respect from others, as well as self-acceptance and self-respect 1. Acceptance without stereotyping or labeling. 2. Respect for persons based on their inherent humanity. 3. Respect for diverse perspectives.	Being listened to. Integration into the community. Respectful interactions.	Lack of self-acceptance and self-deprecation. Rudeness, patronizing attitudes, and diminishment.	Self-stigma may involve reductions in self-esteem and self-efficacy, as well as diminished goal pursuit (46).	Avoidance of contact by members of the general public with individuals diagnosed with a mental disorder (e.g., schizophrenia) due to fear (prejudice) and stereotyped beliefs (stereotypes) that such individuals are dangerous (46).	Stigmatizing attitudes within mental health services; diagnostic overshadowing (physical symptoms misattributed to mental disorder), leading to missed or inaccurate diagnoses (31). Experiences of feeling humiliated and diminished during detailed interviews – often re-traumatizing – conducted by committees deciding on the granting of sickness benefits (138).
Recognition 1. Recognition of one’s own and others’ humanity – fair treatment.	Recognizing others’ shared humanity through attentive listening and genuine appreciation.	Discrimination.	Self-discrimination constitutes a component of self-stigma and refers to behavioral responses to internalized prejudice, such as refraining from pursuing employment (22).	Discrimination, as a component of public stigma, refers to behavioral responses such as avoidance of members of stigmatized groups (46).	Differential responses by mental health professionals operating within institutional norms and practices across mental disorder diagnoses; less stigmatizing and more positive responses toward individuals with depression or anxiety disorders than toward those diagnosed with more highly stigmatized conditions such as schizophrenia or borderline personality disorder (98). Experiences of discrimination in the context of state welfare benefits. Individuals were treated with suspicion and perceived as “scroungers” (139).
Independence 1. Being self-sufficient. 2. Ability to function independently. 3. Ability to make decisions.	Possibility of securing employment. Access to social support programs, including housing and financial support.	Dependence. Paternalism.	An internalized belief endorsing diminished autonomy, paternalistic treatment and reliance on others for decision-making (for example, in mental health care (140).	Individuals with mental health-related diagnoses may be perceived as incapable of independent living or employment in the community (141).	Substantially reduced hiring prospects among applicants disclosing mental health problems, with approximately a 27% lower likelihood of receiving an interview invitation and a 22% lower likelihood of receiving any positive employer response (142). Examination of domestic legislation across 193 countries on marriage rights for people with mental illness: explicit marriage bans in 37% of countries; mental health problems constituting grounds for annulment or nullity in 11% (21 countries) (143).
Ability to seek and use help 1. Willingness to seek help in difficult situations. 2. Seeking support from both formal sources (e.g., healthcare professionals) and informal sources (e.g., family, friends, significant others). 3. A sense that, even when receiving support, one remains a person of worth.	Willingness to accept help. A supportive environment that provides assistance while respecting personal autonomy.	Lack of institutional, personal, or social support. Inappropriate support, including assistance that undermines personal autonomy, disregards individual values, or diminishes a person’s sense of worth.	Refraining from seeking help and avoiding engagement in activities. Avoidance of treatment among people with lived experience due to fear of stigma and anticipated negative reactions (e.g., being perceived as “crazy” or weak, experiencing shame, or concern about others’ judgments) (144).	Blaming and shaming by professionals. Patients reported being blamed and shamed for “deviant” behavior that expressed suffering (e.g., self-harm), with disapproval replacing empathy – these and other experiences may have contributed to disengagement from services and care avoidance (145).	Structural stigma among individuals with mental health and substance use diagnoses, contributing to unmet needs, delays in seeking care, and treatment discontinuation and, consequently, regarded as a fundamental cause of population-level health inequities (144). Socioeconomic bias in treatment selection; blue-collar workers with depression more often offered medication-only care (146).
Activity 1. Activity directed toward other people. 2. Creativity. 3. Defending one’s beliefs and values. 4. Fulfilling one’s responsibilities.	Promotion of prosocial behaviors and economic security that ensure the satisfaction of basic needs.	Social isolation. Reduced engagement in personal and social responsibilities. Criticism or demeaning treatment by others.	Social withdrawal and alienation. Avoidance of close interpersonal contact due to anticipated rejection. Perceived inferiority relative to individuals without mental disorder diagnosis (147). The “Why Try” effect (associated with self-stigma) – a sense of futility in which individuals view themselves as unworthy or incapable of pursuing and achieving personal goals due to a mental disorder (114).	Expressions of social distance toward individuals with diagnosed mental disorders, including unwillingness to have a person with schizophrenia as a neighbor (52).	Reinforcement, through media representations, of the notion that individuals with mental health diagnoses are unsociable (148). Ability-focused academic culture, with mental health difficulties framed as lack of ability, leading students to avoid disclosure and help-seeking, withdraw socially, and experience adverse impacts on academic performance and everyday functioning (149).

Measurement tools for self-stigma, such as the Internalized Stigma of Mental Illness (ISMI) (147) or The Paradox of Self-Stigma Scale (PaSS-24) (150), include items relating to alienation, acceptance of stereotypes, experience of discrimination, or social withdrawal (ISMI), as well as, for example, non-disclosure or avoidance (PaSS-24). Another scale related to self-stigmatization is the Self-Stigma of Mental Illness Scale. The scale consists of four subscales that assess different aspects of self-stigma: awareness, agreement, application and harm to self-esteem (151). Notably, one of its subscales, “Harm to Self- Esteem”, warrants particular attention (152). It aligns with the self-stigmatization model we previously discussed, whose possible consequences include lowered self-esteem and decreased self-efficacy (101, 151). Although this scale (152) and model (46) incorporate harm to self-esteem as the final stage (or consequence) in the self-stigmatization process, empirical findings also suggest that self-esteem and self-efficacy, which may be related to sense of dignity, do not consistently predict self-stigmatization (e.g. in Romania) (153, 154). Moreover, some researchers distinguish sense of dignity from self-worth, emphasizing that sense of dignity encompasses not only self-respect but also the recognition of others’ worth and the commitment to treating their values as equally important as one’s own (121). Thus, self-stigmatization cannot be reduced solely to self-esteem, just as self-esteem is not synonymous with dignity. An interesting example can be found in the study by Sturm and Dellert on a sample of nurses (155). While these conceptualizations of dignity and self-esteem are evidently interconnected and the study reports a relatively strong correlation (r = 0.62), clear distinctions can still be drawn between these two forms of self-characterization, with some items not correlating at all and many showing only weak correlations (155). One study conducted among older adults in Sirjan City has also demonstrated that self-esteem correlates with dignity; however, these constructs also do not fully overlap (156). Some studies (for example, among individuals with Alzheimer’s disease) suggest that dignity consists of multiple components, with autonomy being one of the key elements (157). Self-determination, for example, when deciding about treatment, can be considered an aspect of dignity related to the right of taking risks (158). Concept of dignity involves autonomy and the freedom to make decisions in alignment with one’s beliefs. While this inevitably involves risk, it is also an expression of personal liberty. In addition, Andrejević et al. (159) have demonstrated that knowledge of a victim’s subjective experience of humiliation enhances the perception of offensive treatments as violations of dignity rather than violations of respect. That being said, dignity seems to be more than just the sum of self-esteem, self-efficacy or respect. Consequently, it can be inferred that self-stigmatization and dignity are also distinct constructs that do not fully overlap, even though they may be related. This is consistent with findings from one study that reported only a moderate negative correlation (r = -0.52, p < 0,001) between self-stigma and perceived dignity, suggesting partial but not complete overlap between the measures used in the study (160).

In turn, a sense of self-dignity is associated with positive psychological and social consequences. Among female students, a sense of self-dignity is associated with striving for development, autonomy, undertaking activities to benefit others, interacting socially, and forming social bonds (161). In a reciprocal way, these “consequences” of dignity have the potential to promote a sense of dignity itself. Dignity can combine notions of self-esteem, quality of life, autonomy, well-being, empowerment, hope (162). Self-stigma, in turn, is associated with lower self-esteem and self-efficacy (101), lower sense of quality of life (117, 163, 164), lower hope and empowerment (100).

The sense of dignity can be strengthened through creativity and activity, including activity directed toward other people (124, 125). An example of such activities can be found in LGBTQ activism. The majority of individuals who organized Pride events and engaged in educational initiatives recognized the positive impact of activism, reporting a significant improvement in their overall well-being (165). However, stigma and consequent self-stigma can lead to a “why try” effect, that correlates with diminished personal recovery among people with mental disorder diagnosis (114). People experience a sense of futility, feel undignified, and stop undertaking various activities related to, for example, education, employment, and relationships (114). Larson and Corrigan (166) (p. 525) summarize it in the following way: an individual says, “I’m useless and dangerous so I’m not going to apply for the grain elevator job. They won’t hire me anyway, so why try?”.

The relationships between the concepts of self-stigma and dignity suggest that long-term exposure to stigma has an impact on self-stigmatization and could be related to a lower sense of dignity. Acceptance of stereotypes about people with a diagnosis of mental disorders as the truth about oneself is part of the self-stigma process in the social-cognitive model (102). Accepting the stereotypes leads to devaluation, hence a possible inverse relation with a sense of dignity, which promotes self-valorization, i.e. building a positive self-image, but without treating one’s value as higher than other people’s (121).

## 7 Dignity in the context of mental health care

Individuals with serious mental disorders diagnosis may be particularly vulnerable to dignity violations within healthcare settings, not least because they are more likely to experience hospitalization, including involuntary admission. In psychiatric hospitalization, dignity can be undermined through dehumanization, rights violations, and deprivation of authority. For patients, dignity may be related to being treated with respect and equal recognition (167). Many mental health professionals, in turn, perceive clients as “lacking insight” and as unable to be trusted with treatment-related decision making. It may also partly explain the fact that people who have experienced psychosis do not trust clinicians, because they fear their situation will be misunderstood and/or may lead to involuntary hospitalization (168). Research indicates that between 74% and 80% of individuals admitted involuntarily, as well as 22% to 25% of those admitted voluntarily for mental health reasons, experience a perceived coercion during their hospitalization (169–171). Higher perceived coercion was associated with lower levels of self-dignity among patients in psychiatric ward (59) and stigma-related stress (172). Involuntary hospitalization itself may have harmful effects on the sense of self-worth (173). People with a diagnosis of schizophrenia (or other psychiatric diagnoses) might experience humiliation, being ignored, not being given information about treatment, being forcibly restrained (174).

Dr. Nev Jones’ experience may serve as an illustration of the problem (175). She was diagnosed as a “person with schizophrenia” and treated by healthcare personnel as if every statement she made was delusional. Jones (175) (Suffering in the system) described it the following way: “For years, following program termination, I felt that all I was and had once hoped to be had been reduced to an object to be medicated with more antipsychotics (…)”. This isn’t an isolated case, but points to a common experience of people diagnosed with schizophrenia, involving incarceration institutionalization, and poverty (175). Additionally, involuntary detention in psychiatric care on the grounds of being “a danger to self or others” constitutes a particularly salient challenge for the patient dignity. Individuals, who had experienced this, reported that they felt ignored by healthcare professionals, were unable to participate in treatment decision-making, and were not informed about the treatment plan or the medications prescribed (176). Chambers et al. (176) (p. 6) stressed that “feelings of powerlessness, helplessness and stigma, impinge on the service users’ sense of dignity”. Physical restraint, as used in psychiatric care, also poses a threat to patients’ sense of dignity, because it can restrict access even to basic hygiene facilities (e.g., toileting). Such experiences are commonly described as humiliating by patients (177). However, even in voluntary care, people do not always feel treated with respect. Young adults receiving outpatient mental healthcare emphasized that not everyone is treated with dignity in psychiatric care; however, they also noted that this issue can extends beyond psychiatry to society at large (178).

Women who have experienced sexual violence have highlighted the predominant reliance on a biomedical approach in psychiatric wards: “It was suggested that mental health practitioners – particularly psychiatrists – could be too quick to medicate victim/survivors and label them with a clinical diagnosis, with less focus on addressing the SV (sexual violence) directly. This left the women in this study feeling disempowered and silenced” [ (179 (p. 3152)]. One of the study participants added: “It’s really hard trying to explain this stuff to the services, because they just straight out stereotype you. I could go in and be perfectly sane and just tell them about my life and they’ll label me as ‘insane’ because they just make these assumptions that I’m not well when I am and I’m just reacting healthily to my circumstances and my life during unhealthy times” [ (179) (p. 3147)]. It is also important to note that informal coercion in mental healthcare may also present a threat to dignity. It covers a range of practices (e.g., persuasion, negotiation, interpersonal leverage, inducements, restrictions, deception, threats, and displays of force) (132). According to the definition by Beeri et al. (180), informal coercion is “use of verbal, non-verbal or overt communication patterns, ‘legal’ coercion, deception and manipulation and abuse of power, as well as the enforcement of cultural adaptation and rule conformity, and professional attitudes and skills” (p. 13).

## 8 Discussion

Stigma and consequent self-stigma are an important social problem and may undermine the sense of dignity. Marmot (181) (p. 1020) wrote that “If we cannot define dignity precisely, we will have trouble measuring it. If we cannot measure it, how will we know if we are achieving it?” Defining and operationalizing the concept is crucial if we are to understand and empirically verify, in a quantitative manner, the relationship between stigma and dignity, as qualitative approaches have already highlighted this connection (44).

For the elderly with a diagnosis of schizophrenia, a sense of dignity is both an internal and external experience. It can be eroded by stigma and alienation, and strengthened by relationships (44). Participants in a qualitative study indicated that they consider it to be of great importance to be accepted as a human being by others. This entails that they should not be ignored, ridiculed, and humiliated (44). These behaviors are part of attitudes related to stigmatization (182).

It is crucial to remember that stigmatization is a structural problem (79). Thus, self-stigmatization should be similarly analyzed in the context of wider social processes. In the same way, a sense of dignity is developed when interacting with another person, a group of people or social institutions. Meanwhile, people with diagnoses continue to experience stigma not only from the general population, but also from professionals (183). Respecting dignity could counteract stigma (184) and so minimize internalized stigma.

Additionally, professionals have observed that the process of diagnosis can contribute to the stigmatization of individuals diagnosed with mental disorders (86). People might avoid treatment in order not to receive a stigmatizing label (185). Katherine Ponte (186) who is a mental health advocate and a person who experienced a bipolar disorder diagnosis described her feelings in this way:

“It started by suspecting, feeling that something was not right, but dismissing it, hoping it would pass. The stresses of social injustice, a sexual assault by a friend and classmate, academic stress, career issues, family illness, disappointments to myself and my family. Finally, a shocking diagnosis reached in just a few minutes. I didn’t even know what bipolar was. Nobody took the time to explain it to me. Nobody cared about all of my triggers. They just gave me a label and some meds. Threats of forced medical leave. I was marginalized, isolated, and withdrawn” and added “Self-stigma— the most powerful, most dangerous stigma of all” [ (186), p. 1163].

The experience of stigmatization preceded self-stigmatization. There seems to be no experience of dignity in this story, or, to put it another way, there is a glaring absence of the experience of dignity.

However, when it comes to operationalization, measurement, and research concerning the sense of dignity, the available questionnaires most often apply to people with somatic illnesses, during hospitalization, and the elderly. For this reason, as noted in the introduction, the concept of dignity has been most frequently associated with terminally ill patients, individuals with cancer (42, 43), and older adults (45). Lam et al. (62) reviewed 11 scales, but found that only The Patient Dignity Inventory (PDI), Jacelon Attributed Dignity Scale (JADS), and Inpatient Dignity Scale (IPDS) had acceptable validity and reliability in order to be used to measure dignity among adult patients in palliative care or patients in other hospital wards. JADS (187) was designed with older people in mind and asks about experiences from the past week. The Inpatient Dignity Scale (188) focuses on the behavior of medical personnel in hospitals, the items in the questionnaire concern respect for dignity in the treatment of patients on the ward. In turn, The Patient Dignity Inventory (189) includes questions about symptoms such as dyspnea, nausea and assistance in using the toilet, and medical support. For this reason, they may not be suitable for people with mental disorder diagnoses or to measure sense of dignity understood as a relatively stable trait or a cognitive process.

The conceptual analysis presented here could guide the development of a new tool that could be used in broader contexts, including individuals diagnosed with mental disorders, but also transdiagnostically and perhaps for the general population. The results obtained from such a measurement tool could, for instance, enable the comparison of groups with different diagnoses, demographic characteristics or other minorized groups, helping to determine the direction of the relationship between self-stigma and dignity. The sense of dignity could also be explored as a common factor related to the effectiveness of psychotherapy (190, 191) across diverse therapeutic approaches or other interventions.

Even though many interventions targeting self-stigma are available, few of them have demonstrated effectiveness. The conceptual basis for self-stigma interventions is poorly developed and more attention to conceptualization, measurement tools, and theoretical frameworks is needed (192). For self-stigma related to psychosis, some effective interventions seem to be available, although many with small or moderate effects, high heterogeneity and a risk of bias and very few interventions have a sustained effect (193).

Some studies have confirmed the effectiveness of CBT in reducing self-stigma (194). Interventions based on Group CBT, although they reduced the level of self-stigma, showed no significant differences in comparison to the reduction observed in the control group (195). However, such differences were observed in improving the sense of coherence and resistance to stigma (195). One of the programs aimed at reducing stigma among individuals with various mental health diagnoses was the “Honest, Open, Proud” (HOP) program. HOP is a peer-led group program designed to assist people with mental disorder diagnosis in making independent and dignified decisions about disclosure and managing stigma (196, 197). The HOP program consists of four sessions: (1) Lesson 1 focuses on the benefits and risks of (non-)disclosure, encourages participants to define their goals, and helps them reach a preliminary decision about disclosure in a given situation; (2) Lesson 2 introduces five levels of disclosure, ranging from social withdrawal to active broadcasting, with options such as non-disclosure, selective, and indiscriminate disclosure in between. (3) Lesson 3 guides participants in shaping their personal story and reflecting on lessons learned. (4) Lesson 4 (booster session) reviews participants’ (non-)disclosure attitudes from lesson 3 and discusses any changes in their disclosure experiences or personal stories since then. A meta-analysis found that the HOP program significantly reduced stigma-related stress but had modest, non-significant effects on self-stigma and depression with a small but significant reduction in self-stigma at 3–4 weeks and unknown long-term effects (197). In the case of individuals diagnosed with schizophrenia, researchers attribute these results, to the high level of public stigma (198). Another study also did not confirm the reduction of self-stigma with HOP (196). Still, a RCT conducted with groups of U.S. college students who participated in the booster session demonstrated benefits in self-stigma, disclosure self-efficacy, and resources for coping with stigma (199). HOP programs are based on the assumption that disclosing diagnoses improves well-being and social support and center around the decision to disclose or not. However, the actual role of disclosure (or “empowered non-disclosure”) in the effectiveness of the program is not clear (199). An important component of the program is narrative sharing, where participants develop and share personal stories of their experiences with mental disorder diagnosis, including the onset of mental illness, dark days, recovery, and experiences of overcoming stigma. It is possible, that this narrative-cognitive process itself may enhance sense of dignity, foster a sense of community and agency, and potentially mediate reductions in self-stigma by providing balanced stories of their lives. Thus, it seems that research on the sense of dignity could offer a valuable contribution to the conceptualization and design of these interventions and could be included as a moderating or mediating factor or outcome measure. Although programs aimed at reducing self-stigmatization exist, self-stigma is often preceded by experienced stigma and violations of one’s sense of dignity. Addressing these issues requires systemic and structural changes. Not being subject to coercive treatment, including informal coercion and being involved in care decisions may be crucial factors ensuring perseverance of dignity in mental health-care settings (58, 59). Individuals diagnosed with serious mental health disorders may be particularly vulnerable to treatment practices influenced by public and structural stigma depriving them of their sense of dignity. However, in milder forms, these threats may apply also to patients diagnosed with other disorders. Thus, dignity related interventions should focus not only on patients, but also on clinical staff and other caregivers.

Notably, hospitalization may be related to the sense of dignity, both for patients in general as well as for psychiatric patients. It is also pertinent to explore how hospitalization in a psychiatric ward may differ from other experiences of hospitalization and how it may relate to the sense of dignity. This is particularly relevant, as hospitalized individuals often encounter numerous negative experiences within psychiatric wards (200). Examples include: loss of autonomy, privacy, freedom, power, and choice (200). As previously mentioned, autonomy may be closely linked to the sense of dignity (157, 201). Therefore, research on dignity, its determinants, and its relationship with stigma and self-stigma, both within and beyond psychiatric wards, along with the development of new dignity-related tools, can provide valuable insights and help address an existing gap in the literature. This, in turn, could allow for the development and implementation of various solutions.

For instance, it should be noted that addressing the issue of dignity in the context of mental health may require systemic reforms (202). A patient should not be treated as an individual with problems unrelated to a wider context. Therefore, social and economic factors can be taken into consideration. Social determinants of mental health have a greater impact on overall health than traditional medical risk factors, particularly among individuals with mental disorders diagnosis (203). These determinants include: socioeconomic disadvantage, childhood adversity, housing quality, migration (204). By addressing social, political, and economic determinants, it is possible to promote a sense of dignity by minimizing structural stigma. This can be achieved through equitable healthcare delivery, economic stability, and access to healthy food; implementing changes in legal policies and practices concerning, for example, decision-making capacity in treatment; redefining health and well-being from the perspectives of patients, their families, and communities rather than solely through the healthcare system; and reforming research funding to balance biological and psychosocial studies about etiology (203). It is crucial to recognize the disparities in access to mental health services (205). Clinicians may be biased in selecting treatment due to socioeconomic status, and one study found that blue-collar workers were more likely to be offered only pharmacological treatment for depression (146). This may be related to the nature of diagnostic systems framing distress in terms of individual disorders which leads to their systemic decontextualization, called “disorderism” (206), which may reinforce stigma (207). Psychiatric diagnoses can be inherently stigmatizing. Therefore, it is worth noting that, for example, LGBTQ+ movements not only combated stigmatization but also actively challenged the framing of homosexuality as a mental disorder (208).

Open Dialogue (OD) may be an example of an approach that potentially facilitating a sense of dignity (209, 210). It involves dialogue, respecting autonomy, respecting everyone’s perspective (rather than trying to impose a consensus), avoids asymmetrical relationships, and promotes self-determination (210). Open Dialogue (OD) stresses the need to depathologize human feelings and behaviors – “problematic symptoms” are considered to be normal reactions to a difficult situation (211). This may have a positive effect on the subjective experience of dignity affecting current emotional state and future expectations of an individual (212). These emotional states and expectations, in the long term, will have a positive or negative impact on social functioning, social relations, economic aspects, and the fulfillment of social roles (212). It is possible that the sense of dignity associated with approaches such as OD (213) or Soteria (214, 215) may explain their positive outcomes.

The content and form of anti-stigma efforts require careful reconsideration. It is possible that campaigns promoting biomedical explanations for mental disorders may be ineffective or even harmful, for example by leading to a self-fulfilling prophecy (216–218). Pescosolido et al. (52) question the dominant narrative framing mental problems as “diseases like any other”, and argue that a neurobiological understanding of the causes of mental disorders can contribute to the tendency to stigmatize people labeled as disordered. Solidarity-oriented campaigns (“I stand with you”) are generally rated more positively than normalization campaigns (“You are just like me”), especially by individuals with a diagnosis (219). Educational campaigns using a “Myths and Facts” format have received considerable attention but may be ineffective or even reinforce stigma (220). Celebrity-based campaigns also may have many unintended negative consequences, such as implying that only “exceptional” individuals recover, additionally their effectiveness depends on the match between the celebrity, the content of disclosure, and the intended audience (221, 222). More promising are contact-based interventions, in which people with lived experience lead interactive educational sessions, reflecting the active and social nature of the sense of dignity (223, 224).

Prilleltensky (225) pointed to threats to dignity like materialism, social inequality and injustice. Stigma toward mental disorders may paradoxically be higher among highly educated, male or those with income above the national median compared to participants who were not highly educated, were female, or with lower income, reflecting stigma as a structural tool to maintain social hierarchies (226). Foster and O’Mealey (227) suggest mental health committees should include diverse backgrounds, as higher economic status may be associated with blaming individuals and increased stigma.

Dignity should be present and promoted in health care and mental health care. Respect for dignity is a value that supports health and well-being (228). Therefore, violations of dignity such as stigmatization (and self-stigmatization) should be countered. People diagnosed with a mental disorder asserted that anti-stigma initiatives were not genuinely impactful unless they resulted in structural-level changes through legislative action (229). They considered this to be a crucial indicator of a reduction in stigma and discrimination (229).

Thus, interventions targeting individuals to better manage “self-stigma” may be ineffective in the long-term and actions should not be limited to an individual dimension only. Receiving treatment may result in self-stigma, which disrupts recovery (230) and diagnostic labeling may contribute to self-stigma. It may be valuable to consider alternative approaches to understanding mental distress (231–233) and to explore ways of familiarizing professionals (e.g., psychiatrists, clinical psychologists) with non-categorical frameworks, such as the Power Threat Meaning Framework (PTMF). PTMF highlights the social, cultural, and economic context of distressing experiences and focuses on their meaning and function, rather than solely on identification and classification (231, 233) In line with the idea of diagnostic pluralism, symptom-based categorical systems, such as the DSM, along with specific diagnoses, may serve as a “conversation piece” that invites diverse meanings and perspectives, rather than being treated as fixed classifications (206). Psychoeducation that moves beyond the medical model and biological explanations of mental reified constructs (234) may also contribute to reducing stigma and self-stigma while fostering a sense of dignity. A greater emphasis on the broader life context and living conditions, rather than primarily on presumed biological abnormalities, could be beneficial (235). Additionally, social inequality (236), which may intersect with other forms of stigma – such as the stigma of poverty (237) – could play a significant role in the shaping of dignity of individuals diagnosed with mental health conditions.

## 9 Conclusion

Dignity is a foundational value with significant implications for mental health and psychiatric practice. Psychiatric practice should emphasize the protection of human dignity, which necessitates taking into account the patient’s perspective, combating stigmatization, and fostering relationships based on respect, trust, and individualized care (238). This conceptual analysis argues that dignity is a multidimensional construct that encompasses self-acceptance, self-respect, and the recognition of one’s own values and beliefs, as well as respect for others. This sense of dignity can be nurtured through acceptance, the absence of prejudice, social equity, interpersonal relationships, empathy, trust, and autonomy. Individuals diagnosed with mental disorders, who often contend with both external stigma and internalized stigma, can be particularly vulnerable to experiencing a diminished sense of dignity. Therefore, elucidating the factors that either facilitate or undermine dignity could prove beneficial and it is necessary to develop instruments and conduct empirical studies on the sense of dignity and its relation to mental health diagnoses. Addressing the issue of dignity of psychiatric patients may require a multifaceted strategy that includes alternative frameworks for conceptualizing mental distress, reducing social inequality, enhancing social support networks, and ensuring equitable access to high-quality healthcare. Practical implications for promoting dignity include person-centered care, adherence to ethical standards, the application of approaches such as the Power Threat Meaning Framework or Open Dialogue. Future research should directly examine whether self-stigma and dignity are indeed distinct constructs by using validated measures of both within the same sample. With quantitative research on dignity among individuals diagnosed with mental disorders, it will be possible to propose dignity-enhancing interventions, such as those based on the sharing of personal narratives and life experiences, promoting activities that foster creativity, and autonomy, and providing education for medical staff on behaviors that can be perceived as disrespectful or prejudiced.

This research could provide valuable insights also in the context of other discriminated groups.

Even though we need to stress that this is a conceptual analysis, and the conclusions of the manuscript regarding clinical practice and further research must be treated as preliminary and need to be verified empirically, it seems that promoting a sense of dignity may counteract stigma on social and personal levels. While appropriate tools need to be developed first, future research on the sense of dignity in the context of mental health could facilitate necessary changes in psychiatry and social conditions, ultimately leading to improved treatment outcomes.
